# The Biological Clock Influenced by Burnout, Hormonal Dysregulation and Circadian Misalignment: A Systematic Review

**DOI:** 10.3390/clockssleep7040063

**Published:** 2025-11-03

**Authors:** Alexandru Ungurianu, Virginia Marina

**Affiliations:** 1 Doctoral School of Biomedical Sciences, “Dunărea de Jos” University of Galati, 47 Str. Domnească, 800146 Galati, Romania; alexungurianumm@gmail.com; 2 Medical Department of Occupational Health, Faculty of Medicine and Pharmacy, “Dunărea de Jos” University, 47 Str. Domnească, 800201 Galati, Romania

**Keywords:** burnout, circadian rhythm, cortisol, sleep–wake cycle

## Abstract

Burnout is increasingly recognized as both a psychosocial and a chronobiological disorder characterized by endocrine dysregulation and circadian disruption. It arises from chronic occupational stress and manifests through psychological, physical, and physiological symptoms. Although psychosocial determinants are well established, the biological and chronobiological mechanisms, particularly those involving cortisol and melatonin, remain less explored. This systematic review synthesizes current evidence on hormonal and circadian dysregulation in burnout and complements it with exploratory observational data from healthcare professionals. Peer-reviewed studies evaluating endocrine or circadian biomarkers in individuals with burnout were systematically reviewed. In addition, an exploratory observational analysis was carried out among 195 Romanian clinicians using an adapted Maslach Burnout Inventory. Morning salivary cortisol was measured once at 9 a.m. in a small subsample (n = 26) to provide preliminary physiological data. Because only a single time point was obtained, these values were interpreted as indicative of stress-related activation rather than circadian rhythm. Thirty-seven studies met the inclusion criteria. Across the literature, burnout was associated with altered HPA-axis activity, blunted diurnal cortisol variation, and irregular melatonin secretion related to shift work and disrupted sleep–wake cycles. Complementary exploratory data from our Romanian cohort indicated strong correlations between burnout severity, physical symptoms, and higher morning cortisol values among shift-working clinicians. These findings are preliminary and not representative of full circadian profiles. Burnout should be considered both a psychosocial and a systemic disorder influenced by endocrine and circadian dysregulation. Recognizing alterations in cortisol and melatonin as objective indicators may facilitate earlier detection and inform chronobiological interventions such as optimized scheduling, light exposure management, or melatonin therapy. The observational data presented here is preliminary and intended to generate hypotheses; future research should employ repeated cortisol sampling under controlled Zeitgeber conditions to confirm circadian associations.

## 1. Introduction

Burnout is increasingly recognized not only as a psychosocial syndrome but also as a condition with profound biological underpinnings, including endocrine dysregulation and circadian disruption. Characterized by emotional exhaustion, depersonalization, and reduced personal accomplishment, burnout arises in contexts of chronic occupational stress, especially among healthcare professionals. Recent evidence demonstrates that its pathophysiology extends beyond stress-related psychological symptoms to include alterations in hormonal profiles and sleep–wake regulation. Dysregulation of the hypothalamic–pituitary–adrenal (HPA) axis and suppression of nocturnal melatonin secretion due to night-shift work contribute to the destabilization of circadian homeostasis. Integrative approaches that address occupational, endocrine, and circadian factors are therefore warranted [1,2,3,4].

Burnout syndrome has been extensively studied since its introduction by Freudenberger [5] and the conceptual framework established by Maslach and Jackson [6], who defined the three key dimensions of emotional exhaustion, depersonalization, and reduced personal accomplishment. Healthcare professionals represent one of the populations most vulnerable to burnout due to sustained emotional strain, shift work, and circadian disruption. The current review therefore focused specifically on this group to capture the interplay between occupational exposure, biological stress markers, and circadian misalignment. Over the years, the syndrome has been recognized as a major occupational health issue, with increasing prevalence among physicians, nurses, and other medical professionals, where workload intensity, emotional demands, and frequent night-shift duties are substantial contributing factors [7,8,9,10,11]. The World Health Organization has included burnout as an occupational phenomenon in the International Classification of Diseases (ICD-11), emphasizing its relevance for public health and healthcare systems [8].

At the biological level, burnout is associated with dysregulation of the hypothalamic–pituitary–adrenal (HPA) axis, resulting in abnormal cortisol secretion, ranging from hypercortisolism in early stages to hypocortisolism in chronic stress conditions [11,12,13]. These alterations impair energy metabolism, immune response, and cardiovascular regulation, thereby linking chronic occupational stress to a broad spectrum of physical symptoms and somatic diseases. Complementing cortisol dynamics, melatonin has emerged as a critical biomarker in the context of burnout, given its essential role in circadian synchronization, restorative sleep, and antioxidative defense [4,14,15]. The disruption of melatonin secretion, particularly in night-shift workers, contributes to sleep fragmentation, emotional dysregulation, and increased susceptibility to metabolic and cardiovascular disorders [1,2,3,4].

Several authors have highlighted the systemic character of burnout, demonstrating associations with inflammatory responses, immune suppression, metabolic disturbances, and cardiovascular risk factors [11,12]. Ciobanu et al. reported immune and endocrine alterations in individuals with burnout, while Cadegiani and Kater argued against “adrenal fatigue” as a clinical entity but confirmed robust evidence for cortisol dysregulation as a hallmark of burnout [12,13]. Nash introduced the concept of Dysregulation of Mood, Energy, and Social Rhythms Syndrome (DYMERS), integrating circadian disruption into the explanatory model of stress-related disorders, thereby reinforcing the need to evaluate burnout in relation to chronobiological mechanisms [1].

From a clinical perspective, burnout should therefore be considered a multidimensional syndrome that bridges psychological, physiological, and chronobiological domains. Its consequences extend from impaired professional performance and diminished quality of care to long-term health risks, including cardiovascular morbidity, metabolic disorders, and immune dysfunction. Importantly, healthcare professionals are particularly vulnerable due to the combined effects of occupational stress, high emotional load, and exposure to irregular schedules and night-shift work [1,2,3,4,9,10].

Recent psychophysiological evidence further supports the link between burnout and biological rhythm disturbances. Metlaine et al. described alterations in sleep and biological parameters among professionals with burnout, emphasizing abnormal cortisol and melatonin profiles associated with chronic occupational stress [16]. Likewise, Bagheri Hosseinabadi et al. found that the amplitude and stability of circadian rhythms significantly predicted occupational stress and burnout among irregular-shift nurses [17]. These findings reinforce the need for integrated investigations combining endocrine, behavioral, and chronobiological dimensions of burnout.

To contextualize the present analysis, Table 1 summarizes previous narrative and systematic reviews addressing burnout, hormonal dysregulation, and circadian disruption, including the recent work by Ungur et al. [18]. This synthesis highlights the persistent knowledge gaps that the current review aims to address.

In this context, the present work offers a biopsychosocial review complemented by original empirical data, uniquely integrating evidence on burnout, endocrine dysregulation, and circadian disruption in healthcare professionals, with a particular focus on the interplay between cortisol and melatonin as objective biomarkers of stress and chronobiological misalignment [1,2,3,4].

## 2. Methods

Burnout syndrome is a complex condition influenced by various psychological, physiological, and occupational factors. The latest studies have highlighted its association with hormonal dysregulation, particularly involving the hypothalamic–pituitary–adrenal axis, melatonin secretion, and immune function.

Ghahramani et al. conducted a systematic review and meta-analysis evaluating the occurrence of burnout among healthcare workers during the COVID-19 pandemic. The study synthesized evidence from 52 studies and reported a pooled prevalence of 52%, with nurses and physicians being at the highest risk. The authors identified excessive workload, prolonged exposure to patient suffering, and inadequate support systems as key contributing factors [9]. These findings emphasize the need for institution-driven interventions, such as resilience training and optimized shift scheduling, to mitigate occupational stress and its physiological consequences.

Expanding on these findings, Macaron et al. conducted a systematic review and meta-analysis of physician burnout during the COVID-19 pandemic, encompassing 45 studies. They reported a pooled burnout prevalence of 54.6% (95% CI: 46.7–62.2%). Frontline physicians experienced significantly higher burnout rates compared to second-line colleagues (OR = 1.64, 95% CI: 1.13–2.37) [10]. This study also highlighted chronic stress-related hypothalamic–pituitary–adrenal (HPA) axis dysregulation and altered cortisol dynamics, underscoring the physiological mechanisms that underpin burnout in high-acuity medical environments.

Building on this, Jonsdottir and Sjörs Dahlman further explored endocrine and immunological consequences of burnout, demonstrating that HPA axis dysfunction, immune suppression, and systemic inflammation are prevalent among individuals with burnout. Their study provided physiological evidence showing blunted cortisol responses and elevated inflammatory markers, predisposing affected individuals to metabolic syndrome, cardiovascular disease, and autoimmune disorders [11]. This confirms the notion that burnout extends beyond psychological exhaustion and manifests as a systemic condition with measurable physiological markers.

In a complementary approach, Nash introduced the concept of Dysregulation of Mood, Energy, and Social Rhythms Syndrome (DYMERS), further linking circadian rhythm disturbances to burnout. Investigating 600 healthcare professionals, the study identified irregular sleep patterns and disrupted work schedules as significant worsening factors, impacting melatonin secretion and autonomic nervous system regulation [1]. These findings align with prior research on HPA axis dysregulation and suggest that targeting circadian stability through structured work-rest cycles and light therapy could be an essential intervention for burnout prevention.

Shifting the focus to early-career professionals, Zhou et al. conducted a systematic review and meta-analysis on burnout and stress in trainee physicians. Their findings reinforced that excessive workload, adverse training environments, and insufficient institutional support significantly increase burnout susceptibility [19]. These results highlight the heightened vulnerability of medical trainees to occupational stress and the importance of early interventions, such as mentoring, workload regulation, and resilience training, during postgraduate education. When considered alongside previous studies linking burnout to hypothalamic–pituitary–adrenal (HPA) axis dysregulation, these findings strengthen the case for integrative strategies that address both organizational and physiological risk factors in training environments.

Extending beyond healthcare, Glandorf et al. investigated burnout among athletes in a systematic review and meta-analysis, reinforcing the systemic impact of chronic stress. The study, which synthesized evidence from 54 studies including nearly 14,000 athletes, reported that athlete burnout is consistently associated with higher risk of depression, anxiety, and decreased positive mental health outcomes, with mixed evidence regarding physical health consequences. Some studies highlighted increased vulnerability to musculoskeletal complaints and physiological stress markers, suggesting that sustained physical and psychological strain may contribute to endocrine dysregulation and impaired recovery [20]. These parallels between occupational and athletic burnout emphasize the need for multidisciplinary interventions, spanning both psychological support and endocrinology-based strategies for stress regulation.

Furthermore, Rotenstein et al. highlighted significant variability in burnout prevalence among physicians, ranging from 0% to 80.5%, largely due to inconsistencies in assessment tools and diagnostic criteria. Their study stressed the importance of standardized burnout measurement scales to enhance comparability across research. Furthermore, they found that high burnout levels correlate with increased medical errors, decreased job satisfaction, and elevated cardiovascular disease risk, in a way confirming findings from prior studies on HPA axis dysregulation and inflammatory responses [21]. These results reinforce the need for system-wide policy changes, structured workload redistribution, and long-term stress biomarker monitoring.

Our study was performed on 195 medical staff working in hospitals and clinics across Galați city, Romania. The cohort size was determined pragmatically, based on voluntary participation during occupational-health evaluations, rather than on a priori power analysis. Given the exploratory design, this sample was deemed sufficient to illustrate descriptive associations between stress, circadian disruption, and physiological indicators without inferential testing. The respondents are 125 women and 70 males, with a professional experience ranging from 2 to 40 years. Also, the questionnaire was presented only to operating healthcare professionals, whilst retired staff or those with incomplete responses were excluded. We focused on medical personnel that work on a fixed schedule from 9 to 17:00 and clinicians that perform night shifts or on-call shifts. The data collected provides insights into demographic characteristics, professional background, health-related metrics, and burnout dimensions.

Collectively, these studies illustrate the systemic nature of burnout, emphasizing its far-reaching effects beyond the psychological domain. The integration of endocrinological, immunological, and occupational health perspectives underscores the necessity for a multidisciplinary approach to mitigating burnout. Understanding these physiological mechanisms can inform targeted interventions, including stress management strategies, hormonal regulation therapies, and institutional policies aimed at reducing occupational stress. The findings from these studies serve as a foundational basis for further exploration into the physiological groundwork of burnout and the development of evidence-based preventive measures, reinforcing the need for a multidisciplinary approach in mitigating its widespread impact (Table 2).

Studies published before 2000 were excluded to ensure methodological comparability with contemporary burnout definitions, modern healthcare work patterns (e.g., current shift systems), and standardized biomarker assays, thereby improving interpretability across included studies.

Data was extracted based on study design, sample size, hormonal biomarkers analyzed, and primary findings. The Newcastle–Ottawa Scale (NOS) was used to assess the quality of included non-randomized studies [14,15,16,17]. It evaluates three principal domains: selection of study groups, comparability of cohorts, and outcome assessment. Each study was rated on an eight-point scale, with higher scores indicating greater methodological rigor. Two independent reviewers performed the assessment, resolving discrepancies through discussion or consultation with a third evaluator. The NOS framework ensured that only studies with robust methodology and minimal risk of bias were included in this review.

## 3. Results and Discussion

This systematic review followed the Preferred Reporting Items for Systematic Reviews and Meta-Analyses (PRISMA 2020) guidelines and was prospectively registered in PROSPERO (ID CRD420251045486) [22].

**Figure 1:**
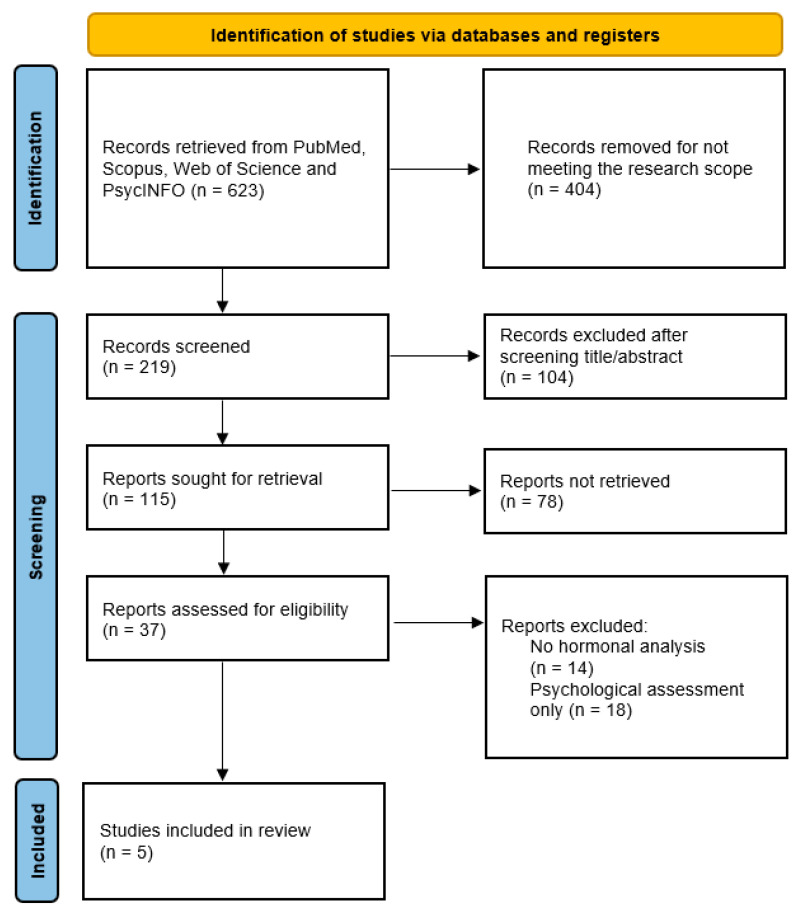
PRISMA flow diagram for the identification, screening, and inclusion of studies. The 78 records listed as “not retrieved” represent duplicate entries across databases, inaccessible full-texts, or abstracts outside the scope of burnout, endocrine biomarkers, or circadian-rhythm research that were excluded during the eligibility-screening stage.

Also, a PRISMA flow diagram summarizing the study identification, screening, and inclusion steps is available both in Figure 1.

Electronic searches were performed in PubMed, Scopus, Web of Science, and PsycINFO on 10 May 2025. The following Boolean combination of terms was used: (burnout AND (cortisol OR melatonin OR “circadian rhythm” OR “sleep–wake cycle”) AND (healthcare OR nurses OR physicians)).

Filters were applied for human studies, English language, and the publication years 2000–2025. Studies published before 2000 were excluded to maintain methodological consistency and ensure comparability with contemporary diagnostic criteria, healthcare environments, and occupational-stress frameworks.

The included studies are presented in Table 3.

### Excluded Studies

As indicated by the PRISMA flow diagram, a total of 32 studies were excluded following the eligibility assessment process. The reasons for exclusion were primarily methodological limitations, which affected the alignment of these studies with the physiological and endocrinological scope of the current systematic review. Specifically, studies were excluded if they did not incorporate hormonal biomarker analyses, consequently failing to provide measurable physiological correlations necessary for inclusion. Additionally, studies that exclusively emphasized psychological parameters without evaluating physiological markers were also excluded, as this review explicitly focused on hormonal dysregulation. Further exclusions resulted from insufficient sample sizes, as limited participant numbers compromised the reliability and general applicability of findings. Studies relying on secondary data (such as narrative reviews or theoretical analyses) and individual case studies were also excluded to ensure the robustness and empirical grounding of the synthesized evidence.

This thorough selection of literature ensured that only studies meeting strict methodological rigor and directly relevant to the physiological dimensions of burnout were included, thereby enhancing the validity and applicability of the review’s conclusions.

**Figure 2:**
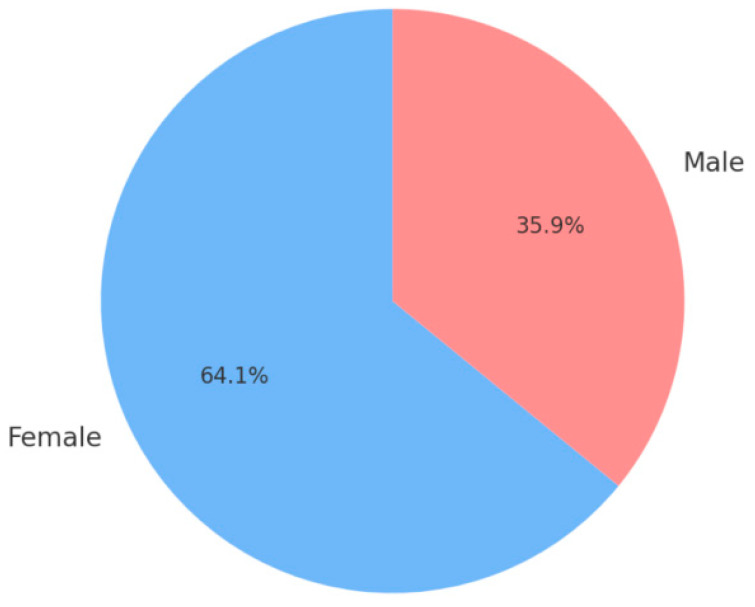
Gender distribution of the questionnaires’ respondents.

The scope of the present analysis was to compare the previous studies with the inquiry that was conducted with the aim to investigate and forecast the prevalence and severity of burnout among healthcare professionals by examining it core dimensions such as emotional exhaustion, depersonalization, diminished personal accomplishment, but including also associated physical health symptoms. Additionally, the research sought to identify demographic and occupational factors potentially contributing to these outcomes. The study specifically targeted healthcare professionals employed in Romanian medical settings, including hospitals and outpatient clinics, and comprised physicians, nurses, and other clinical support personnel and Figure 2 illustrates the gender distribution of the respondents.

Emphasizing both psychological and physiological dimensions, the report aimed to deepen the understanding of occupational burnout within the Romanian healthcare context and provide insights relevant to preventive interventions and clinical practice.

This analysis expands upon traditional burnout assessment frameworks by enhancing the Maslach Burnout Inventory by including targeted items assessing physical symptoms frequently reported among healthcare professionals experiencing chronic occupational stress. Particularly, additional questions focused on somatic complaints such as persistent headaches, musculoskeletal pain, and gastrointestinal disturbances were integrated to capture the physiological dimension of burnout broadly.

**Figure 3:**
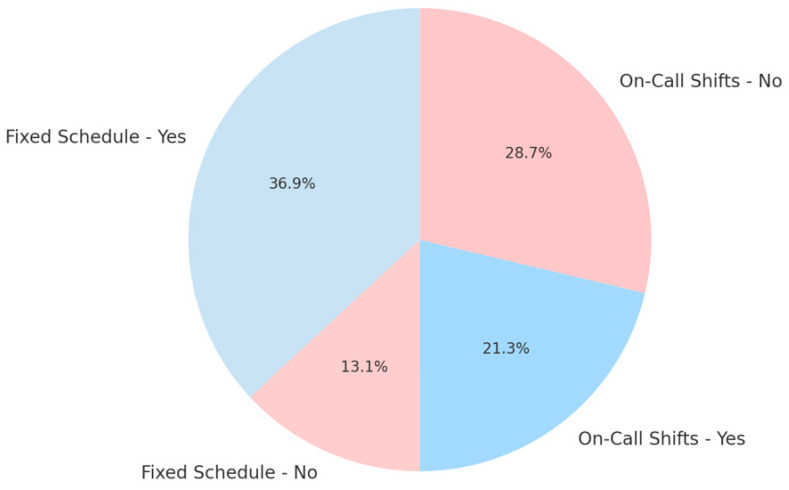
Work schedules and occupational patterns among healthcare professionals.

Recognizing the critical role occupational factors play in the development of burnout, this study also examined the impact of work schedule structures as shown in Figure 3 mainly night shifts and irregular work patterns which significantly influence circadian rhythm stability.

The fixed schedule is determined by the 8 a.m.–4 p.m. or 9 a.m.–3 p.m. program, varying upon request. On-call shifts are determined by duty shifts and night shifts.

Previous literature consistently demonstrates that irregular shift patterns disrupt the physiological regulation of sleep–wake cycles and hormone secretion, especially melatonin and cortisol, exacerbating stress responses and contributing to both physical symptoms and overall burnout severity.

**Figure 4:**
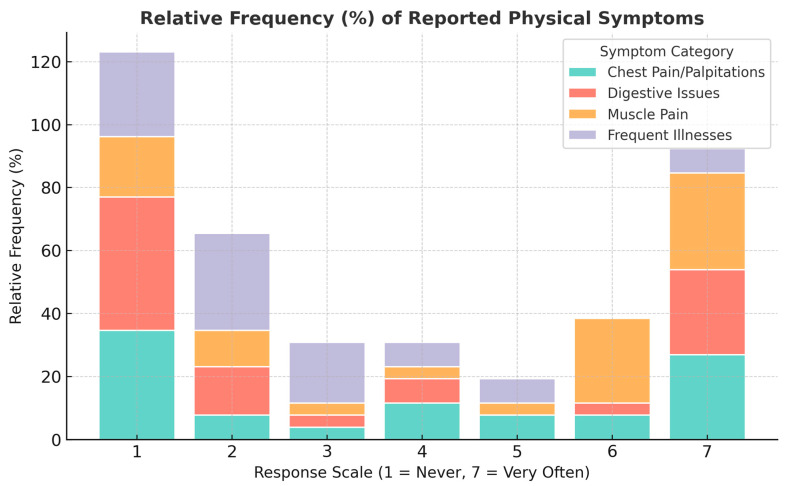
Relative frequency of reported physical symptoms among respondents. Note. Each bar represents the relative frequency (%) of responses for 195 healthcare professionals per symptom category (chest pain/palpitations, digestive issues, muscle pain, and frequent illnesses) across the 7-point response scale (1 = Never, 7 = Very Often). Percentages were calculated separately for each symptom to illustrate the proportional distribution of reported physical manifestations of burnout.

Figure 4 illustrates the prevalence of various physical symptoms—chest pain/palpitations, digestive issues, muscle pain, and frequent illnesses—reported by healthcare personnel. The 7-point response scale (where 1 = Never and 7 = Very Often) allows for a clear assessment of how frequently these symptoms occur among respondents. The data highlights that a significant proportion of participants experience these symptoms, particularly at lower to moderate frequencies.

By incorporating these occupational and physiological dimensions, the study aims to provide an integrated perspective that advises the development of more effective preventive measures, workplace interventions, and clinical practices. Understanding the relationship between demanding work schedules and the manifestation of physical symptoms is essential in tailoring interventions that support the well-being of healthcare personnel exposed to high occupational stress.

In a separate exploratory component, we examined associations between self-reported stress-related symptoms and morning cortisol levels in a subsample of 26 healthcare professionals. Cortisol was collected once at 9 a.m.; therefore, this analysis does not represent a circadian or diurnal profile but a single-time-point snapshot of the physiological stress response. Individual cortisol rhythms differ substantially across people, and determining a true circadian pattern would require several equally spaced samples over a 24-h period and strict control of external Zeitgebers (time cues such as light exposure, food intake, and activity). Because only a single morning sample was collected, no baseline correction or normalization was applied; cortisol values are reported as raw, unadjusted concentrations intended solely for descriptive comparison within this exploratory analysis. As such, this component is explicitly described as preliminary and descriptive, intended only to illustrate potential physiological correlations of burnout within the broader context of the systematic review.

The examination made in Table 4 provides critical insights into the multidimensional environment of burnout, emphasizing notable associations between demographic, occupational, and physiological factors.

A particularly strong correlation (0.81) exists between burnout scores and the presence of physical symptoms, including cardiac manifestations (palpitations or chest pain), gastrointestinal disturbances (digestive problems), cephalic symptoms (headaches), musculoskeletal pain (particularly in the neck, shoulders, or back) and compromised immune function (frequent illnesses). These symptoms suggest that chronic occupational stress and burnout extend beyond psychological distress, translating into measurable physiological disturbances. These disturbances frequently result from persistent activation and dysregulation of stress-responsive neuroendocrine pathways, notably involving the hypothalamic–pituitary–adrenal axis and the sympathetic-adrenomedullary system. Chronic activation of these pathways results in sustained elevations in cortisol and catecholamines, triggering cardiovascular, gastrointestinal, musculoskeletal, and immune dysfunctions.

Furthermore, occupational scheduling emerges as an important variable influencing burnout. A clear positive correlation (0.26) was observed between irregular work schedules and burnout severity, emphasizing the destabilizing effect of on-call and rotating shifts. Conversely, regular schedules demonstrated a slight protective influence (−0.15), suggesting that circadian stability contributes to lower burnout scores. These findings reinforce the role of chronobiological disruption as a mediator between occupational demands and stress-related exhaustion.

**Figure 5:**
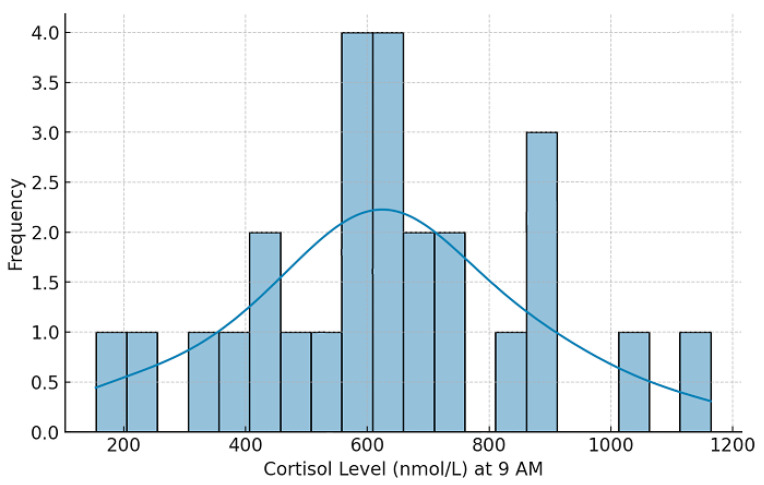
Distribution of morning cortisol levels (9 a.m.).

Figure 5 shows the distribution of morning cortisol values obtained from 26 healthcare professionals with irregular work schedules and on-call duties. Because only a single morning sample was collected at 9 a.m., these data cannot be used to infer circadian or diurnal variation. The results therefore illustrate relative differences between participants rather than true rhythmic patterns. Individual cortisol secretion profiles vary substantially; consequently, this exploratory analysis should be interpreted as a preliminary indication of stress-related physiological activation rather than a characterization of circadian function.

The analysis revealed that certain stress symptoms appear more closely linked to elevated cortisol levels, particularly palpitations, headaches, and muscle pain. Respondents experiencing frequent palpitations or chest pain exhibited a tendency toward higher cortisol levels, suggesting a connection between chronic stress and cardiovascular responses. Similarly, individuals reporting severe muscle pain or frequent headaches were more likely to have elevated cortisol, reinforcing the well-documented link between stress and musculoskeletal tension, as well as stress-induced headaches. On the contrary, the relationship between cortisol levels and digestive issues or frequent illnesses was less pronounced. While some respondents with high cortisol reported frequent digestive problems, the overall correlation was weaker, indicating that other factors may also contribute to gastrointestinal distress. Additionally, although chronic stress is known to impact immune function, no strong direct correlation emerged between cortisol levels and the frequency of illnesses, though individual cases suggest that prolonged occupational stress may still play a role.

These findings emphasize the physiological burden experienced by healthcare professionals exposed to irregular work schedules and on-call shifts. The observed associations between heightened cortisol levels and specific stress symptoms highlight the urgent need for targeted workplace interventions, including stress management programs and preventive health strategies, to support the well-being of professionals in high-stress environments.

Such disruption adversely affects melatonin production, a hormone centrally involved in the regulation of sleep–wake cycles and circadian homeostasis. Irregular shift patterns disturb the nocturnal secretion of melatonin, compromising restorative sleep, impairing cognitive and emotional resilience, and increasing susceptibility to both psychological and somatic manifestations of stress. This disruption is often exacerbated by irregular exposure to environmental cues such as light, meal timing, and physical activity patterns, leading to further hormonal dysregulation, metabolic disturbances, and heightened stress responsiveness.

In contrast, fixed scheduling exhibits a modest negative correlation with burnout (−0.15), suggesting a potential protective effect through maintenance of stable circadian alignment. A consistent work schedule promotes regular patterns of melatonin secretion, enhancing sleep quality and physiological recovery, thus potentially mitigating burnout severity. Regularity in occupational patterns not only stabilizes melatonin secretion but also facilitates optimal functioning of the endocrine system more broadly, including cortisol and other regulatory hormones. This circadian stability provides physiological resilience against the adverse effects of chronic occupational stress.

The obvious associations outlined in this table underscore the significance of integrating circadian biology and endocrine assessments within burnout evaluations. Recognizing the physiological bedrocks of burnout offers an essential foundation for implementing targeted clinical interventions, such as optimized shift scheduling, structured rest periods, melatonin supplementation, lifestyle modifications, and workplace policy reforms aimed at stabilizing circadian rhythms. Moreover, addressing these physiological disturbances can enhance clinical outcomes, reduce occupational stress levels, and significantly improve the overall well-being and health of healthcare professionals.

Our exploratory cortisol findings reflect morning stress reactivity rather than circadian dynamics, as only one sampling point was obtained. Hence, these results should be interpreted as indicative of physiological stress load, not of intrinsic circadian rhythmicity. The pattern observed nevertheless aligns with existing medical evidence demonstrating that hormonal dysregulation—particularly involving the hypothalamic–pituitary–adrenal (HPA) axis—plays a pivotal role in the pathophysiology of burnout. The strong correlations between burnout severity and physical symptoms emphasize that chronic occupational stress extends far beyond psychological distress, producing measurable physiological consequences. These somatic manifestations—such as cardiovascular irregularities (palpitations, chest pain), gastrointestinal dysfunction, headaches, musculoskeletal discomfort, and immune impairment—are consistent with stress-related HPA-axis activation described in prior studies [11,12].

Human field investigations rarely achieve complete control of Zeitgebers—external “time givers” such as light exposure, meal timing, and physical activity—that synchronize endogenous circadian rhythms to the 24-h day. Only constant-routine protocols can isolate intrinsic biological rhythms, minimizing such cues to reveal the internal phase and amplitude of circadian processes [23]. Such control was beyond the scope of this exploratory design, which aimed instead to illustrate the biological plausibility of stress-related endocrine activation in healthcare professionals. Nevertheless, burnout cannot be fully understood without integrating the role of circadian disruption, particularly in professions where irregular work schedules and night shifts are pervasive. Our results, showing a moderate but meaningful positive correlation between burnout severity and on-call or night-shift schedules, align with prior evidence linking occupational scheduling to circadian misalignment and melatonin dysregulation [1,2,3,4]. Circadian misalignment disrupts melatonin secretion and impairs restorative sleep, thereby exacerbating emotional exhaustion, diminishing resilience, and aggravating physiological stress responses. Consistent with [4], exposure to light at atypical hours and irregular sleep–wake timing suppresses nocturnal melatonin and desynchronize biological rhythms, amplifying downstream metabolic and mood effects. On the contrary, our finding of a modest negative correlation between burnout and fixed work schedules supports the protective role of circadian stability. This protective effect is consistent with evidence that structured routines stabilize melatonin secretion and support physiological recovery, reducing vulnerability to stress-related endocrine disturbances [1,2,3,4].

The interplay between cortisol and melatonin represents a central mechanism in the chronobiology of burnout. Cortisol follows a diurnal pattern, peaking in the morning and decreasing throughout the day, while melatonin secretion peaks at night to promote restorative sleep. Chronic stress and irregular schedules disrupt these rhythms, resulting in both HPA axis dysregulation and suppression or desynchronization of melatonin secretion. These disruptions propagate downstream effects on metabolic health, immune regulation, and cardiovascular stability [9,10,24,25]. Nash’s concept of Dysregulation of Mood, Energy, and Social Rhythms Syndrome (DYMERS) is particularly relevant, as it integrates the impact of circadian misalignment on both psychological functioning and biological homeostasis [1]. This framework suggests that burnout cannot be adequately addressed without considering the synchrony—or lack thereof—between biological clocks and occupational demands.

The evidence also highlights the need to differentiate burnout from contested constructs such as “adrenal fatigue.” As Cadegiani and Kater emphasized, there is insufficient empirical support for adrenal fatigue as a distinct entity; however, there is robust evidence of cortisol dysregulation associated with chronic stress and burnout [13]. Our data support this nuanced perspective: while adrenal fatigue as a diagnostic label is unsubstantiated, the measurable alterations in cortisol secretion patterns observed in burnout represent real physiological changes with clinical implications. Similarly, studies by Jonsdottir and Sjörs Dahlman demonstrated immune suppression and systemic inflammation among burnout patients, highlighting that the syndrome is systemic, not limited to psychological exhaustion [11].

An additional contribution of our study is the integration of physical symptomatology into burnout assessment. The strong correlations we observed between burnout severity and somatic complaints (r = 0.81 for physical symptoms, r = 0.90 for emotional exhaustion) emphasize the psychosomatic character of occupational stress. These findings align with Ciobanu et al., who identified neuroendocrine alterations linked to immune dysfunction in burnout, reinforcing the need for multidimensional assessment tools that capture both psychological and physiological aspects of the syndrome [12].

The role of night-shift work and circadian misalignment therefore deserves particular attention. Prior literature shows that irregular shift patterns destabilize melatonin and cortisol rhythms, impairing sleep quality, cognitive functioning, and emotional stability [26,27,28,29]. Our findings confirm that irregular schedules significantly increase burnout severity, while fixed or regular schedules provide resilience by stabilizing circadian alignment. This has important implications for workplace policy: interventions that reduce night-shift frequency, increase schedule predictability, manage light exposure, and incorporate structured rest periods could act as protective chronobiological strategies against burnout [1,2,3,4].

Clinical strategies for managing burnout should therefore extend beyond traditional psychological interventions to include chronobiological approaches. Preliminary evidence suggests that melatonin supplementation may improve sleep initiation, stabilize circadian rhythms, and mitigate stress-induced physiological arousal; however, these findings remain tentative and require confirmation in larger controlled studies. Likewise, early findings indicate that adaptogenic herbs such as Rhodiola rosea and Withaniasomnifera (ashwagandha) may modulate cortisol secretion and enhance stress resilience, but current evidence is still limited and should be interpreted with caution [14,15]. When combined with organizational interventions—such as workload redistribution, resilience training, and optimized scheduling, these strategies may collectively offer a more comprehensive and biologically grounded approach to mitigating burnout risk.

Despite these insights, several limitations should be acknowledged. The heterogeneity of included studies, in terms of design, biomarkers assessed, and burnout definitions, complicates direct comparisons. Melatonin measurement remains methodologically challenging due to its pulsatile secretion and sensitivity to environmental cues such as light and meal timing. Our own empirical contribution relied primarily on cortisol assays, while melatonin dysregulation was inferred through circadian context. Future research should integrate real-time biomarker monitoring (e.g., wearable actigraphy combined with salivary cortisol and melatonin profiling) to capture the dynamic interplay of stress and circadian regulation. Additionally, longitudinal studies are needed to clarify causal relationships between circadian disruption and burnout progression.

In sum, this discussion underscores that burnout is best conceptualized as both a psychosocial and a chronobiological disorder. Cortisol and melatonin act as complementary biomarkers: cortisol reflecting HPA axis activation and stress reactivity, melatonin representing circadian integrity and restorative capacity. Their combined dysregulation explains why chronic occupational stress produces such profound physical consequences. Recognizing this dual framework has significant clinical and organizational implications, opening the door to integrated interventions that stabilize circadian rhythms, normalize endocrine function, and address occupational stressors at their root.

## 4. Conclusions

The present review and exploratory empirical findings underscore that burnout must be understood as both a psychosocial syndrome and a systemic disorder underpinned by endocrine and circadian dysregulation. Burnout manifests not only through emotional exhaustion, depersonalization, and reduced personal accomplishment, but also through measurable physiological alterations, including abnormal cortisol dynamics (as indicated by single morning cortisol levels in our exploratory sample, which cannot establish full circadian variation), disrupted melatonin secretion, and the emergence of somatic symptoms such as cardiovascular dysfunction, musculoskeletal pain, gastrointestinal complaints, headaches, and impaired immune function [11,12]. Cortisol data was obtained from a limited exploratory subsample (n = 26), which restricts inferential power but provides descriptive insight into stress-related endocrine variability. These systemic effects reflect the dual disruption of the hypothalamic–pituitary–adrenal (HPA) axis and circadian regulation, confirming that burnout extends beyond psychological exhaustion into biological dysfunction. These observational results should be regarded as preliminary and hypothesis-generating; future research must employ repeated cortisol sampling under controlled Zeitgeber conditions to characterize true circadian alterations [23].

Our findings demonstrate that irregular work schedules and night-shift duties significantly amplify burnout severity by destabilizing circadian rhythms. These observations are consistent with prior literature linking night-shift work to circadian misalignment, melatonin suppression, and impaired sleep–wake cycles [1,2,3,4]. Conversely, fixed or predictable schedules appear to offer partial protection, stabilizing circadian homeostasis and reducing physiological vulnerability to stress. This emphasizes the importance of organizational strategies that minimize shift irregularity, optimize work–rest cycles, and support circadian alignment in healthcare professionals.

Melatonin emerges as a particularly valuable biomarker and potential therapeutic agent. Beyond its established role as a “chronobiotic” regulating circadian phase, melatonin exerts antioxidative, anti-inflammatory, and neuroprotective properties.

Incorporating melatonin into burnout research and intervention frameworks could improve early detection of circadian misalignment and enable chronotherapeutic approaches. Alongside melatonin, adaptogenic herbs such as *Rhodiola rosea* and *Withaniasomnifera* have shown promise in modulating cortisol secretion and enhancing stress resilience, complementing more established organizational and psychological interventions [14,15].

The integration of physical symptomatology into burnout assessment, as demonstrated in our dataset, highlights the psychosomatic character of occupational stress and the necessity of multidimensional diagnostic tools. This aligns with previous studies that emphasized the neuroendocrine and immune alterations associated with burnout [11,12], reinforcing that both psychological and biological domains must be considered when developing diagnostic and preventive strategies.

Despite advances, significant gaps remain. The heterogeneity of definitions and biomarker methodologies continues to hinder cross-study comparisons. Melatonin measurement remains challenging due to its pulsatile and light-sensitive secretion, while cortisol assays vary in precision and interpretability across studies [25]. Future research should prioritize real-time monitoring of cortisol and melatonin rhythms, combined with wearable sleep–wake tracking, to better capture the dynamics of stress and circadian disruption. Moreover, longitudinal multicenter studies are needed to validate biomarkers and establish causal pathways linking occupational stress, circadian misalignment, and health outcomes.

In conclusion, burnout should be reframed not only as an occupational and psychological phenomenon but also as a chronobiological disorder. Recognizing the dual involvement of cortisol and melatonin as complementary biomarkers provides a more comprehensive understanding of the syndrome and opens new opportunities for integrative interventions. These include organizational reforms to stabilize work schedules, chronotherapeutic strategies such as light therapy and melatonin supplementation, and targeted stress-reduction approaches that restore both endocrine and circadian balance. Such a multidisciplinary approach is essential for protecting healthcare professionals from the escalating burden of burnout and for ensuring the long-term sustainability of healthcare systems.

## Figures and Tables

**Table 1:** Summary of previous reviews addressing burnout, hormonal dysregulation, and circadian disruption.

Study Title	Authors	Study Type	Sample Size	Key Findings
Dysregulation of Mood, Energy, and Social Rhythms Syndrome (DYMERS) [1]	Nash, C. (2025)	Scoping Review	600 healthcare workers	Irregular social and biological rhythms contribute to burnout symptoms, with disrupted sleep and circadian misalignment.
Burnout Among Healthcare Workers During the COVID-19 Pandemic [9]	Ghahramani, S. et al. (2021)	Systematic Review & Meta-Analysis	52 studies	Burnout prevalence was 52%, with nurses and physicians at the highest risk. Contributing factors included workload and emotional exhaustion.
A Systematic Review and Meta-Analysis on Burnout in Physicians During the COVID-19 Pandemic: A Hidden Healthcare Crisis [10]	Macaron, M.M. et al. (2023)	Systematic Review & Meta-Analysis	45 studies	Burnout prevalence was 54.6% (95% CI: 46.7–62.2%), with frontline physicians more affected than second-line colleagues (OR = 1.64, 95% CI: 1.13–2.37).
Endocrine and Immunological Aspects of Burnout [11]	Jonsdottir, I.H., & Sjörs Dahlman, A. (2019)	Narrative Review	Multiple studies reviewed	HPA axis dysfunction and immune suppression are linked to burnout, leading to increased physical health risks.
A Narrative Review of Burnout Syndrome in Medical Personnel [18]	Ungur, A.P. et al. (2024)	Narrative Review	Multiple studies reviewed	Synthesizes evidence on burnout among medical personnel and emphasizes hormonal and psychosomatic mechanisms relevant to clinical practice.
Factors Associated with Burnout and Stress in Trainee Physicians [19]	Zhou, A.Y. et al. (2020)	Systematic Review & Meta-Analysis	30 studies	Workload, training environment, and inadequate support systems were identified as the main factors influencing burnout among trainee physicians.
Mental and physical health outcomes of burnout in athletes [20]	Glandorf, H.L. et al. (2023)	Systematic Review & Meta-Analysis	54 studies; 13,976 athletes	Athlete burnout associated with increased negative mental health, decreased positive mental health; mixed evidence on physical health outcomes
Prevalence of Burnout Among Physicians [21]	Rotenstein, L.S. et al. (2018)	Systematic Review	40 studies	Burnout prevalence varied widely (0% to 80.5%), highlighting the need for standardized assessment methods.

**Table 2:** Eligibility Criteria.

Criterion	Inclusion Criteria	Exclusion Criteria
Study Design	Empirical studies, systematic reviews, and meta-analyses	Non-peer-reviewed sources, conference abstracts, opinion pieces
Population	Individuals diagnosed with burnout, healthcare professionals	Studies without hormonal biomarker analysis
Outcomes	Cortisol, melatonin, immune markers, metabolic indicators	Psychological outcomes only
Language	English or widely accessible languages	Non-English studies without translation
Publication Year	2000–2024	Studies published before 2000

**Table 3:** List of included studies.

Study	Sample Size	Biomarkers Assessed	Key Findings
Jonsdottir & Sjörs Dahlman (2019) [11]	1200	Cortisol, HPA axis	Burnout associated with blunted cortisol response
Ciobanu et al. (2021) [12]	800	Cortisol, immune markers	Neuroendocrine alterations linked to immune dysfunction
Cadegiani & Kater (2016) [13]	750	Cortisol, metabolic markers	No direct evidence for adrenal fatigue but strong metabolic impact
Nash (2025) [1]	600	Melatonin, circadian rhythms	Shift work disrupts melatonin secretion in burnout cases
Kakiashvili et al. (2013) [3]	1000	Cardiovascular markers	Burnout increases cardiovascular disease risk

**Table 4:** Summary of Key Correlations between Burnout and Physical Symptoms.

Factors	Burnout Score Correlation	Interpretation
Physical Symptoms	0.81 (strong positive)	Increased physical symptoms are strongly associated with burnout severity, indicating significant physiological impacts (cardiac, digestive, headaches, musculoskeletal, immune).
Emotional Exhaustion	0.90 (strong positive)	Emotional exhaustion is a primary contributor to burnout, closely related to physiological stress.
Depersonalization	0.82 (strong positive)	Higher depersonalization levels correlate strongly with increased burnout, emphasizing psychological distancing as a stress-coping mechanism.
On-call Shifts (Irregular schedule)	0.26 (moderate positive)	Irregular shift work contributes notably to burnout, likely due to disruptions in circadian rhythm and melatonin secretion.
Fixed Schedule (Regular schedule)	−0.15 (negative correlation)	Regular schedules show a protective effect, suggesting that circadian stability may mitigate burnout.
Experience Years	−0.17 (negative correlation)	Greater experience slightly reduces burnout risk, feasibly through improved coping mechanisms.
BMI	−0.02 (no significant correlation)	BMI does not significantly correlate with burnout in this analysis, indicating limited immediate metabolic impact within this context.
Gender	−0.03 (no significant correlation)	Gender showed negligible correlation with burnout severity, suggesting burnout prevalence is similar across genders in this context.

Note: Because of the exploratory design and the limited subsample (n = 26), confidence intervals and *p* values were not calculated. The reported correlation coefficients are descriptive and intended to illustrate relative associations.

## Data Availability

The dataset supporting the findings of this study is available on request from the corresponding authors.

## Abbreviations

a.m.	anterior meridian
p.m.	post meridian
DYMERS	Dysregulation of Mood, Energy, and Social Rhythms Syndrome
HPA	Hypothalamus–Pituitary–Adrenal
MBI	Maslach Burnout Inventory
NOS	Newcastle–Ottawa Scale
SAM	Sympathetic-Adrenomedullary System
